# "No Touch to Touch": Percutaneous Sclerotherapy of a Giant Hepatic Cavernous Hemangioma

**DOI:** 10.7759/cureus.111240

**Published:** 2026-06-21

**Authors:** Renato Abu Hana, Carlos I Gonzalez Baerga, Grit A Adler, Vinicius Fornazari

**Affiliations:** 1 Radiology, University of Florida College of Medicine - Jacksonville, Jacksonville, USA

**Keywords:** bleomycin, ct, lipiodol, sclerotherapy, ultrasound guided

## Abstract

Cavernous hemangiomas were originally taught as "no touch lesions" as they were benign, hypervascular, venous malformations. They are commonly found in the liver. Patients typically present asymptomatically, and these hemangiomas can be found incidentally on imaging as solitary or multiple lesions. A 42-year-old male patient arrived at our institution after a motor vehicle collision, and a giant hemangioma was found incidentally on imaging with a classic feature of peripheral nodular enhancement on arterial phase computed tomography (CT) imaging. However, the patient continued to report right upper quadrant pain. Given the patient's age and size of the lesion, percutaneous sclerotherapy using bleomycin and lipiodol was performed. At the two-week follow-up, the patient denied fever, nausea, vomiting, or pain. At six months, the patient was asymptomatic, and follow-up CT angiography (CTA) demonstrated an approximately 50% reduction in lesion size. Therefore, percutaneous sclerotherapy is an effective, low-risk, and technically straightforward treatment alternative to the traditional intraarterial approach, resulting in significant lesion size reduction and meaningful symptomatic relief with a favorable safety profile.

## Introduction

Cavernous hemangiomas are among the most common benign neoplasms of the liver with an incidence between 0.4% and 20% and with female predilection. They are benign vascular hamartomas composed of endothelial-lined vascular spaces filled with slow-flowing blood. These lesions are often diagnosed incidentally on imaging via ultrasound or computed tomography (CT). Although most patients are asymptomatic, some may present with abdominal pain and swelling [1].

Small- to medium-sized hemangiomas, generally less than 4 cm in diameter, rarely require intervention. Giant hemangiomas, defined as greater than 5 cm in diameter, carry a higher risk of complications such as abdominal pain, hemorrhage, jaundice, and coagulopathy. When symptomatic, current treatment options include surgical resection, radiofrequency ablation (RFA), transarterial embolization (TAE), and percutaneous sclerotherapy (PS) [2,3].

The intraarterial use of bleomycin and lipiodol emulsion for the treatment of giant hepatic hemangiomas has been previously studied with a high technical success rate and a clinical success rate of 80.6% [4]. Yuan et al. demonstrated a reduction in tumor size by approximately over 50% [5].

The purpose of this case report is to demonstrate that PS with bleomycin and lipiodol is a simple, quick, and low-risk procedure alternative that can achieve significant lesion reduction and symptomatic relief in patients with giant hepatic cavernous hemangiomas.

## Case presentation

A 42-year-old male patient presented to the emergency department (ED) following a motor vehicle collision complaining of epigastric pain. CT angiography (CTA) of the chest, abdomen, and pelvis with venous and delayed phases was performed per trauma protocol and revealed a giant hepatic hemangioma measuring approximately 9.4 × 7.1 × 10.6 cm with classic peripheral nodular enhancement on arterial phase imaging (Figure 1). No intraabdominal traumatic injuries were identified.

**Figure 1:**
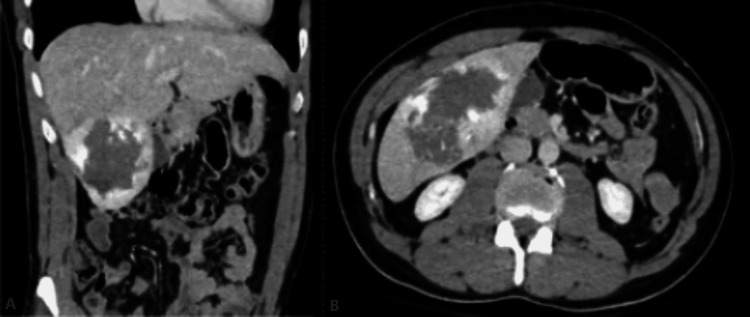
Preoperative computed tomography (A) Coronal and (B) axial computed tomography angiography images of the chest, abdomen, and pelvis (trauma protocol, venous and delayed phases) demonstrating a large hypodense hepatic mass with peripheral nodular enhancement on arterial phase imaging, measuring 9.4 × 7.1 × 10.6 cm, consistent with a giant cavernous hemangioma.

During the admission, the patient reported a long-term history of mild right upper quadrant pain predating the accident, which had worsened following the collision. Given the incidentally discovered giant hemangioma and the patient's symptoms, a decision was made to follow up with the patient in the interventional radiology clinic one month later. At the consult, the patient reported that he remains with abdominal pain and agrees to proceed with PS using bleomycin and lipiodol. Pre-procedure laboratory evaluation was performed, including hepatic function, and was completely normal, with no relevant findings.

Procedure technique

After formal consent was obtained according to the institution's protocol, the patient was prepared and draped.

The procedure was performed under moderate sedation with intravenous fentanyl and midazolam, with local anesthesia achieved using lidocaine infiltration at the skin entry site. Under ultrasound guidance, a 22-gauge Chiba needle (PAJUNK® GmbH Medizintechnologie, Geisingen, Germany) was advanced into the center of the hemangioma (Figure 2). Iodine contrast was injected showing no evidence of hepatic artery, portal vein, hepatic vein, or biliary duct opacification, confirming safe intracavitary positioning and excluding vascular or biliary communication prior to sclerosant delivery. A mixture of 45 units of bleomycin in 10 mL of Lipiodol® Ultra Fluid (Guerbet, Aulnay-sous-Bois, France) was carefully injected into the lesion under continuous fluoroscopic monitoring. The needle was repositioned two times for better tumor coverage. The patient was discharged two hours later reporting no pain (0/10 on the Numeric Pain Rating Scale (NPRS)) nor procedural complications.

**Figure 2:**
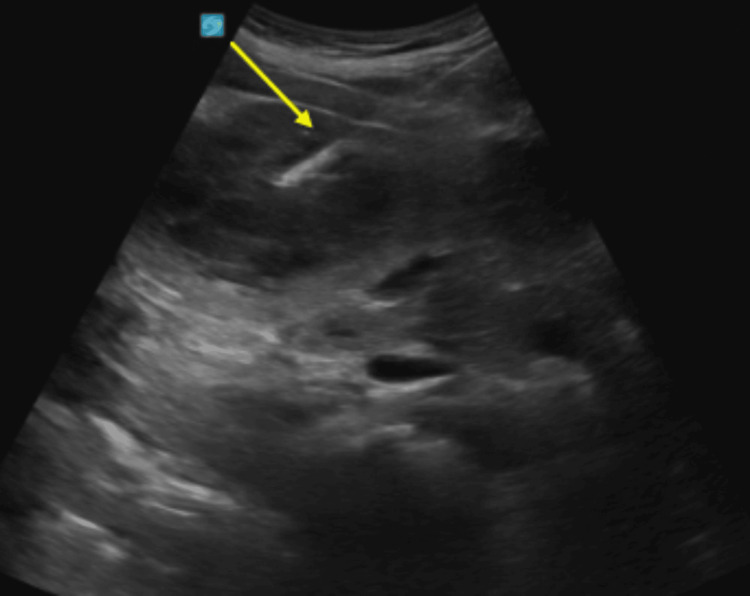
Intraoperative ultrasound Intraprocedural ultrasound image of the right hepatic lobe demonstrating a hypoechoic mass consistent with a giant cavernous hemangioma. A 22-gauge Chiba needle (yellow arrow) is seen advancing into the lesion under real-time sonographic guidance prior to sclerosant injection.

At the two-week post-procedure follow-up, the patient reported significant improvement in right upper quadrant pain, reporting occasional residual discomfort of 1/10 on the NPRS, managed with over-the-counter acetaminophen. No post-procedural complications were identified. Laboratory values obtained post-procedure remained within normal limits, with no significant changes compared to pre-procedure baseline (Table 1). The patient was seen again at the six-month follow-up with repeat multiphase CT of the abdomen (arterial and portal venous phases) demonstrating an interval decrease in size of the giant hemangioma, now measuring 5.8 × 4.6 × 5.8 cm (Figure 3), representing an approximately 50% reduction in maximum linear diameter compared to baseline dimensions of 9.4 × 7.1 × 10.6 cm. It should be noted that linear diameter was used as the primary response surrogate, as volumetric measurements were not available at both time points; this represents a known limitation of size-based response assessment in single-lesion case reports. Laboratory findings at six months remained stable and within normal range. The next follow-up imaging is scheduled for one year after the procedure.

**Table 1:** Patient lab values Laboratory values obtained pre-procedure and post-procedure with normal reference ranges. CBC: complete blood count; WBC: white blood cells; INR: international normalized ratio; AST: aspartate aminotransferase; ALT (SGPT): alanine aminotransferase (serum glutamic-pyruvic transaminase)

Laboratory test	Units	Reference range	Pre-procedure	Post-procedure
CBC				
WBC	×10³/µL	4.5-11.0	10.37	9.80
Hemoglobin	g/dL	13.5-17.5	13.4	13.6
Hematocrit	%	41-53	42.2	42.8
Platelet count	×10³/µL	150-400	327	318
Coagulation				
INR	ratio	0.8-1.2	1.0	1.0
Liver function tests				
AST	U/L	10-40	17	19
ALT (SGPT)	U/L	7-56	10	12
Alkaline phosphatase	U/L	44-147	82	78
Total bilirubin	mg/dL	0.1-1.2	0.7	0.8
Albumin	g/dL	3.5-5.0	4.0	4.1

**Figure 3:**
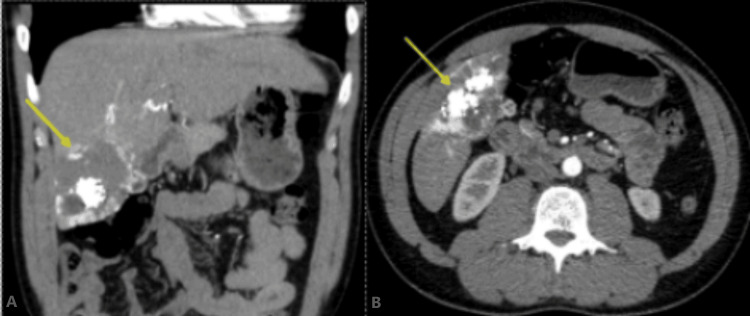
Postoperative computed tomography Six-month follow-up computed tomography of the abdomen in (A) coronal and (B) axial planes demonstrating significant interval decrease in hemangioma size measuring 5.8 × 4.6 × 5.8 cm, with an approximately 50% reduction compared to baseline imaging. Note the dense central area (yellow arrow) related to lipiodol deposition.

## Discussion

Cavernous hemangiomas are endothelium-lined vascular spaces perfused by slow-flowing blood and represent the most common benign hepatic neoplasm. The majority of cases are managed conservatively given their benign natural history. However, giant hemangiomas, generally defined as those exceeding 5 cm in diameter, can cause progressive symptoms including abdominal pain, early satiety, and nausea and may rarely be complicated by spontaneous rupture or the Kasabach-Merritt phenomenon, a life-threatening coagulopathy. When treatment is indicated, current options include surgical resection, RFA, and minimally invasive image-guided approaches such as TAE and PS.

Surgical resection, while definitive, carries significant procedural risks including hemorrhage, infection, and prolonged recovery. It is often not feasible depending on lesion location, proximity to major hepatic vessels, or patient comorbidities [6]. 

Percutaneous RFA has been reported for smaller symptomatic hemangiomas; however, complication rates increase substantially with lesions greater than 10 cm in diameter, and the procedure typically requires high doses of sedation or general anesthesia, limiting its applicability in giant lesions [2,6].

TAE using a bleomycin and lipiodol emulsion has been one of the most widely studied intraarterial approaches for giant hepatic hemangiomas. Yuan et al. retrospectively evaluated 241 patients who underwent superselective TAE and demonstrated greater than 50% reduction in maximum tumor diameter at six, 12, 36, and 60 months of follow-up, with high technical and clinical success rates [5]. Despite its efficacy, TAE is associated with well-documented risks inherent to the arterial access and catheterization required, including embolization of non-target vessels, biloma and hepatic abscess formation, post-embolization syndrome, and hepatic artery dissection or spasm. Additionally, TAE requires arterial access, catheter-based navigation, and often longer procedural times, adding cost and complexity compared to a purely percutaneous approach [5].

PS avoids arterial catheterization entirely, relying instead on direct image-guided needle access into the hemangioma. Various sclerosing agents have been described, including ethanol, detergents, and hyperosmotic solutions; however, bleomycin in combination with lipiodol has emerged as the preferred agent combination given its favorable safety profile and efficacy [3]. Bleomycin exerts its sclerosing effect through the non-specific inhibition and destruction of endothelial cells via DNA damage and the induction of an inflammatory response, ultimately leading to endothelial-mesenchymal transition, tissue ischemia, fibrosis, and lesion shrinkage [7,8]. Critically, the slow-flowing blood within hepatic hemangiomas allows for prolonged contact time between bleomycin and the endothelial lining, potentiating its sclerosing effect and contributing to progressive lesion involution [3]. Lipiodol, an iodinated ethyl ester of poppy seed oil, is selected as the drug carrier rather than aqueous contrast or saline for several important reasons. As an oily embolic agent, lipiodol is selectively retained within low-flow vascular spaces, prolonging the contact time of bleomycin with the target endothelium and enhancing drug delivery to the lesion periphery. Its radiopacity allows real-time fluoroscopic monitoring of the injection, enabling the operator to confirm intracavitary distribution and detect any inadvertent communication with adjacent vascular structures before the sclerosant is delivered [3]. Regarding dosing, published protocols for percutaneous hepatic hemangioma sclerotherapy establish 45-60 units as the recommended single-session dose of bleomycin mixed with 10 mL of lipiodol, injected slowly under fluoroscopic guidance after confirmatory contrast injection [7].

This range is defined by the risk of bleomycin-induced pulmonary toxicity, specifically pulmonary fibrosis, which has been reported at high cumulative lifetime doses exceeding 300-400 mg, and per-session doses are therefore maintained well below this ceiling [7,8]. Importantly, bleomycin dosing in this context is not calculated based on lesion volume. Published data demonstrate that pretreatment lesion volume does not correlate significantly with treatment response, as the sclerosing effect depends on drug-endothelium contact time rather than total drug volume delivered [4]. Broader intratumoral coverage is instead achieved by needle repositioning during the procedure, as was performed in this case. In the present case, 45 units was administered, within the established safe range, with the needle repositioned twice to optimize lesion coverage. Given the satisfactory reduction in size and complete symptom resolution at six months, a second treatment session was not required; however, repeat sclerotherapy remains a viable option should symptoms recur or lesion size fail to regress further on long-term follow-up.

A recent systematic review and meta-analysis published in CardioVascular and Interventional Radiology directly comparing PS and transarterial chemoembolization (TACE) for giant hepatic hemangiomas found that both techniques achieved 100% technical success. Radiological success was comparable between groups (TACE: 81.9%; PS: 81.29%), and while TACE showed slightly higher clinical success (99.9% vs. 89.73%), this difference was not statistically significant (p = 0.15). Importantly, major complication rates were similarly low in both groups, with PS demonstrating a lower systemic complication profile [9].

Regarding patient selection, surgical resection is no longer considered a first-line option for most symptomatic giant hepatic hemangiomas, given its significant perioperative risks, and is generally reserved for lesions with diagnostic uncertainty, rapid growth, or failure of minimally invasive approaches. Among image-guided options, both TAE and PS are appropriate first-line treatments. TAE may be preferred for lesions with a dominant arterial supply amenable to superselective catheterization or when multifocal disease requires the treatment of multiple lesions simultaneously. PS is particularly well-suited for patients with accessible unilobar lesions, those in whom arterial access is undesirable due to coagulopathy or vascular anatomy, or when a simpler outpatient procedure under moderate sedation is preferred. In this case, PS was selected over TAE given the lesion's accessibility, the patient's otherwise good health, and the goal of minimizing procedural complexity while achieving equivalent clinical outcomes.

Although no complications were observed in this case, operators should be aware of several potential adverse events associated with PS of hepatic hemangiomas. Access-related complications include hepatic hemorrhage and subcapsular hematoma. Inadvertent injury to adjacent structures may result in vascular or biliary fistula formation. The most consequential risk specific to this technique is non-target delivery of the bleomycin-lipiodol emulsion into the portal venous or hepatic venous system. Should the sclerosant drain into the hepatic veins and reach the pulmonary circulation, two serious pulmonary complications may result, that is, lipoid pneumonia from lipiodol embolization of the pulmonary vasculature and bleomycin-induced pulmonary toxicity, which can range from subclinical pneumonitis to severe acute respiratory distress syndrome (ARDS) and, in rare cases, may be fatal [7]. This underscores the critical importance of the pre-injection contrast test, which must confirm the absence of any vascular or biliary communication before the sclerosant is administered.

## Conclusions

PS with bleomycin and lipiodol represents a simple, safe, and effective minimally invasive option for symptomatic giant hepatic cavernous hemangiomas. Performed under moderate sedation without the need for arterial access, it avoids the procedural complexity and complication risks associated with surgical resection or TAE while achieving significant lesion size reduction and durable symptomatic relief. This case supports PS as a viable first-line interventional option that can be performed in an outpatient setting. Future prospective studies with larger cohorts and standardized follow-up protocols are warranted to further define long-term outcomes and optimal dosing strategies.
